# Inhibition of K^+^ Channels Affects the Target Cell Killing Potential of CAR T Cells

**DOI:** 10.3390/cancers16223750

**Published:** 2024-11-06

**Authors:** Ghofrane Medyouni, Orsolya Vörös, Vivien Jusztus, György Panyi, György Vereb, Árpád Szöőr, Péter Hajdu

**Affiliations:** 1 Department of Biophysics and Cell Biology, Faculty of Medicine, University of Debrecen, 4032 Debrecen, Hungary; medyouni.ghofrane@med.unideb.hu (G.M.); orsolyavoros89@gmail.com (O.V.); jusztus.vivien@med.unideb.hu (V.J.); panyi@med.unideb.hu (G.P.); gvereb2020@gmail.com (G.V.); akuka@med.unideb.hu (Á.S.); 2 Faculty of Pharmacy, University of Debrecen, 4032 Debrecen, Hungary; 3 Division of Dental Biochemistry, Faculty of Dentistry, University of Debrecen, 4032 Debrecen, Hungary

**Keywords:** CAR T cells, ion channels, Kv1.3, KCa3.1, CRAC, cancer, immunotherapy

## Abstract

Ion channels of T cells (Kv1.3, KCa3.1, and CRAC) participate in the regulation of activation and effector functions via modulation of the Ca^2+^-dependent pathway. T cells expressing chimeric antigen receptors (CAR T cells) showed a remarkable role in anti-tumor therapy, especially in the treatment of chemotherapy-resistant liquid cancers. Nevertheless, many challenges remain to be overcome to improve the treatment for solid tumors. In this study, we assessed the expression and role of ion channels in CAR T cells. We found that HER2-specific CAR T cells had higher KCa3.1 conductance compared to the non-transduced (NT, control) cells, which was more prominent in the CD8^+^ population (CD4^+^ cell also showed elevation). Conversely, the Kv1.3 expression level was the same for all cell types (CD4^+^, CD8^+^, CAR, and NT). Single-cell Ca^2+^ imaging revealed that thapsigargin-induced SOCE via CRAC is suppressed in CD8^+^ CAR T cells, unlike for CD4^+^ and CD8^+^ NT cells. To dissect the functional role of Kv1.3 and KCa3.1, we used specific antagonists (Kv1.3: Vm24; KCa3.1: TRAM-34): the target cell elimination capacity of the CD8^+^ CAR T cells was improved either by blocking KCa3.1 or Kv1.3. These results imply that ion channels could be a target in CAR T cell immunotherapy elaboration.

## Simple Summary

CAR T cells have been a breakthrough in the field of immunotherapy in the past few years. More than six CAR T cell therapies are already approved by the FDA. However, many side effects remain to be solved as well as the low efficacy in solid tumors. The aim of our study was to investigate the role of ion channels Kv1.3 and KCa3.1 in the function of CAR T cells. We revealed differences in the level of expression of these channels, and modulation of the channels’ function could facilitate target cell elimination. These findings highlight the significance of ion channel targeting in CAR T cell therapy.

## 1. Introduction

Cancer immunotherapy partly relies on the reprogramming of host immune cells to eliminate cancer cells. Genetic modification of T cells to express chimeric antigen receptors (CARs) has been a breakthrough in cell-mediated therapy [1,2]. CAR-expressing T cells (CAR T cells) to target a specific antigen have shown great potential to upgrade the approach in the fight against cancer. A CAR is composed of an extracellular domain for tumor antigen recognition (a single-chain variable fragment, scFv) formed by the variable heavy (V_H_) and light (V_L_) chains of an immunoglobulin. This is linked to one or more intracellular signaling domains that mediate T cell activation via the transmembrane segment of a T cell plasma membrane molecule (e.g., CD28 and CD8). CAR T cells are capable of specifically recognizing their target through the scFv causing activation in a major histocompatibility complex (MHC)-independent manner [3]. The first generation of these CAR T cells had CD3ζ alone as a signaling domain. The second generation incorporated a costimulatory domain, e.g., 4-1BB/CD28. Furthermore, the third CAR generation contains multiple costimulatory signaling domains to induce a proinflammatory environment [4]. CAR T cell therapy represents a major improvement in personalized cancer treatment. It has been approved by the FDA for the treatment of some hematological malignancies, such as large B cell lymphoma (LBCL) [5]. However, despite its success, many challenges remain to improve the efficacy and safety of this therapy due to the exhaustion of the CAR T cells, toxicity effect, and limited response in the case of solid tumors [6]. Studies reported that many patients do not respond to the treatment and suffer from neurotoxicity and cytokine release syndrome [7]. Moreover, the challenging suppressive tumor microenvironment makes the survival and proper function of this therapy difficult [8].

Ion channels in T cells participate in the regulation of multiple functions such as activation, proliferation, cytokine production, differentiation, and migration via the Ca^2+^-dependent pathway. Human T cells express mainly two types of K^+^ channels: the voltage-gated Kv1.3 and the calcium-activated KCa3.1 [9]. These two channels are similar regarding their high selectivity for K^+^ but different in their gating mechanism and pharmacology. Kv1.3 channels are activated by depolarization of the membrane, whereas KCa3.1 is activated upon an increase in the cytosolic Ca^2+^ concentration [10]. These two K^+^ channels maintain the negative membrane potential and therefore sustain the driving force for a Ca^2+^ influx through CRAC (calcium release-activated Ca^2+^) channels [11].

Upon the antigen binding to the T cell receptor, PLCγ generates IP_3_, which causes the release of the calcium from the endoplasmic reticulum (ER) through IP_3_ receptors. In turn, the stromal interaction protein, STIM1, relocates and directly associates with the N- and C-termini of the ORAI1 (CRAC formed by pore-forming ORAI1 and STIM1) and activates the CRAC channel. This leads to the second phase of Ca^2+^-level elevation in the cytosol. Subsequently, the calcineurin-NFAT pathway is activated leading to gene transcription having a prominent role in T cells [12]. It was reported that the deletion or mutation of STIM1 or ORAI1 in human T cells induced the abolishment of the activation and reduced cytokine production (IL-2, IL-4, INF-γ, etc.) [13]. This results in increased susceptibility to severe infections with pathogens as well as autoimmune inflammations [10]. Inhibition of any of these above-mentioned channels by specific blockers can impair the activation and proliferation of T cells or other effector functions [14]. In autoimmune diseases such as rheumatoid arthritis (RA) and multiple sclerosis (MS), Kv1.3 blockers can hamper the autoreactive T cell functions, making them a therapeutic target [15,16].

T cells are major players in tumor immunity: various T cell subtypes are recruited into the tumor site. However, anti-tumor functions of CD8^+^ T cells are downregulated due to the defensive mechanisms developed by the tumor cells. The failure to eliminate the cancer cells could be partially attributed to the suppressor cells like myeloid suppressor cells (MSC) as well as the regulatory T cells (T_reg_) [17]. In addition, cancer cells produce different molecules (e.g., adenosine) because of necrosis and upregulated metabolism that modifies the tumoral ionic milieu (increased K^+^ and H^+^ concentration). This impairs the cytotoxic T cells’ function [18].

The function of CAR T cells should be fully understood to enhance the efficiency of the tumor cell killing as well as the tumor infiltration and persistence. Recent studies showed the correlation between the killing efficiency of CAR T cells and the cytokine/chemokine profile in the case of solid tumors [19]. However, so far, there are no data about the role of ion channels in the CAR T cells. In this study, we determined the expression of these three channels in a third-generation CAR T cell directed against HER2 antigen expressed on the surface of breast cancer cells. In addition, we analyzed the function of two potassium channels in the target cell elimination capacity. We suppose our results can facilitate the design of novel CAR T cell generations, which can be more effective in solid tumor eradication with less harmful side effects.

## 2. Materials and Methods

### 2.1. Cells and Culture Conditions

HEK 293T packaging cells, the triple-negative human breast cancer cell line MDA-MB-468 (abbreviated MDA in the main text), and N87 human gastric cancer cell lines were purchased from the American Type Culture Collection (ATCC, Manassas, VA, USA). The cells were cultured in Dulbecco’s Modified Eagle Medium (DMEM) supplemented with 2 mmol/l GlutaMAX and 10% Fetal Calf Serum (FCS) and antibiotics. The JIMT-1 human breast cancer cell line was established in the laboratory of Cancer Biology, University of Tampere, Finland [20]. These cells were cultured in a 1:1 ratio of Ham’s F-12 and DMEM supplemented with 20% FCS, 300 U/L insulin, 2 mmol/l GlutaMAX, and antibiotics. The primary human T cells and CAR T cells were cultured in the RPMI (Roswell Park Memorial Institute) medium supplemented with 2 mmol/l GlutaMAX and 10% FCS and antibiotics.

All the cells and cell lines were maintained in a humidified atmosphere containing 5% CO_2_ at 37 °C and were routinely checked for the absence of mycoplasma contamination by PCR. MDA.ffLuc, JIMT-1.ffLuc, and N87.ffLuc were generated by single-cell cloning of the MDA-MB-468, JIMT-1, and N87 cell lines, respectively, after transduction with a retrovirus encoding eGFP.ffLUC to express an enhanced green fluorescent protein/firefly luciferase fusion gene [21].

### 2.2. Retrovirus Production and Transduction of T Cells

The RD114-pseudotyped retroviral particles were generated by transient transfection of the HEK 293T cells with the HER2-specific CAR-encoding pSFG retroviral vectors, the Peg-Pam-e plasmid coding MoMLV gag-pol, and the pMax.RD114 plasmid using jetPrime transfection reagent (Polyplus, Illkirch, France) [22,23]. The backbone of the HER2-specific chimeric antigen receptors was composed of the IgG heavy chain signal peptide; the HER2-specific single-chain variable fragment FRP5; the IgG1 short hinge; the transmembrane region of human CD28; and the cytoplasmic region of human CD3 zeta, with CD28 and 41BB providing intracellular costimulation. Supernatants containing the retrovirus were collected after 48 h.

The experiments were carried out on human samples by the Declaration of Helsinki and approved by the Regional and Institutional Committee for Research Ethics (RKEB.5378/2019). To generate CAR T cells, human peripheral blood mononuclear cells were isolated by Ficoll gradient centrifugation and stimulated in non-tissue culture 24-well plates precoated with 1 µg/mL OKT3 (Thermo Fischer, Waltham, MA, USA) and anti-CD28 (R&D Systems, Minneapolis, MN, USA) antibodies. On day 2, human interleukin-7 (IL-7; 10 ng/mL) and human interleukin-15 (IL-15; 5 ng/mL) (Miltenyi Biotec, Bergisch Gladbach, Germany) were added to cultures. The T cells were transduced with retroviral particles on RetroNectin-coated (Takara, Kusatsu, Japan) plates on day 3 in the presence of IL-7 (10 ng/mL) and IL-15 (5 ng/mL). The expansion of the T cells was subsequently supported with IL-7 and IL-15. The OKT3/CD28-activated non-transduced (NT) T cells were expanded with IL-7 and IL-15 using the same protocol. Following 48 h incubation, the cells were used for further experiments [21].

### 2.3. Cytotoxicity Assay

In order to investigate the specific cytotoxic activity of CAR T cells, we used a luciferase-based cytotoxicity assay. MDA, JIMT-1, and N87 cells expressing eGFP/ffLUC were seeded into 96-well flat bottom plates at a concentration of 3 × 10^4^ cells/well in triplicates. Then, 24 h later, different effector cells were added at a 0.5:1 or 1:1 effector-to-tumor cell ratio. The wells without effector cells served as untreated controls. After 24 h, luciferase activity was determined using a luciferase assay kit according to the manufacturer’s instructions (Promega, Madison, WI, USA) and a Synergy HT luminometer (BioTek, Winooski, VE, USA).

### 2.4. Electrophysiology

CD3^+^ CAR/NT T cells were plated onto a poly-l-lysine-coated 35 mm petri dish, while CD4 and CD8 CAR T and NT cells were adhered to a petri dish using the antibody adhesion protocol as described before [24]. Then, the cells were washed with extracellular solution containing 145 mM Na-aspartate, 5 mM KCl, 2.5 mM CaCl_2_, 1.0 mM MgCl_2_, 5.5 mM glucose, and 10 mM HEPES (pH 7.4). The Kv1.3 and KCa3.1 currents in the CAR T cells were measured in a whole-cell voltage-clamp configuration with an Axopatch 200B amplifier (Molecular Devices, Sunnyvale, CA, USA). The pipette solution contained 145 mM K-aspartate, 10 mM K_2_EGTA, 8.5 mM CaCl_2,_ 2 mM MgCl_2,_ and 10 mM HEPES (pH 7.2, 290–310 mOsm, and 1µM free Ca^2+^ concentration). The currents were recorded by 200 ms ramp depolarization from −120 to + 50 mV from a holding potential of −70 mV at every 10 s. KCa3.1 conduction was defined as the ratio of the linear fraction of the macroscopic current slope and the slope of the voltage-ramp stimulus after subtraction of the leak current, *G_KCa3.1_(nS)* = $I_{slope}\left(\frac{pA}{ms}\right)/V_{slope}\left(\frac{V}{ms}\right)$. The slope conductance was measured between −100 and −60 mV to avoid contamination by the Kv1.3 current. For the Kv1.3 current, it was determined from the same ramp protocol at 50 mV after subtraction of the KCa3.1 current extrapolated by linear regression. The conductance for each channel type was normalized to whole-cell capacitance (which is proportional to the channel number per unit area) and was applied to define the membrane expression level.

### 2.5. Intracellular Ca^2+^- Measurement in CAR T Cells

To test the CRAC-dependent Ca^2+^ signaling, first, the cells were labeled using CD4-Alexa488 or CD8-Alexa488 (Biolegend, San Diego, CA, USA). The cells were suspended in 10% BSA in PBS for 30 min on ice, then washed 2 times with PBS, and then resuspended up in phenol-red free media. Afterwards, the cells were plated in poly-L-lysine-coated glass bottom Petri dishes. Next, we stained the cells with 1µM of the calcium-sensitive dye FURA-2 acetoxymethyl ester (Thermo Fisher Scientific, Budapest, Hungary) dissolved in DMSO.

The loaded cells were incubated for 30 min at 37 °C in phenol-red free RPMI solution (Sigma-Aldrich Ltd., Budapest, Hungary), supplemented with 1% FBS, 2 mM L-glutamine, 1 mM Na-pyruvate, and 200 units penicillin/streptomycin.

The CAR T cells as well as the control cells were washed with 2 mM Ca^2+^ solution (143.3 mM NaCl, 4.7 mM KCl, 10 mM HEPES, 5.5 mM glucose, 2 mM CaCl_2_, 1 mM MgCl_2_, and pH 7.35) at the first stage. To perform this experiment, we used an inverted fluorescence microscope NIKON ECLIPSE Ts2R combined with a VisiChrome High-Speed Polychromator (Visitron Systems GmbH, Puchheim, Germany). The temperature was set to 37 °C during the whole measurement. Afterwards, the CAR/NT T cells were perfused with 2 mM Ca^2+^ solution, 0 mM Ca^2+^ solution (143.3 mM NaCl, 4.7 mM KCl,10 mM Hepes, 5.5 mM glucose, 1 mM MgCl_2_, 0.1 mM EGTA, and pH 7.35) and then we applied 0 mM Ca^2+^ solution along with 1 µM Thapsigargin (TG) (Thermo Fisher Scientific) in order to deplete the ER Ca^2+^ stores.

Following store depletion, we added 2 mM Ca^2+^ extracellular solution in the presence of 1 µM TG to induce the intracellular Ca^2+^ elevation via SOCE (store-operated calcium entry).

FURA-2 dual excitation and emission were accomplished using 340 nm and 380 nm excitation filters and a 510 nm emission filter. The digital images (200 ms exposure) were recorded with a PCO Edge 4.2 sCMOS Camera at 10 s intervals. The imaging data acquisition and analyses were accomplished using VisiView^®^ 4.0.0.11 imaging software. 

### 2.6. Statistical Analysis

For our statistical analysis, we used the GraphPad Prism software version 8.0.1. One-way ANOVA or ANOVA tests were performed for the multiple comparison. As for the two groups’ comparison, we conducted the unpaired t test or Mann–Whitney test. The value of *p* < 0.05 was set as a significant difference. The results were shown as the mean ± standard error of the mean (SEM).

## 3. Results

### 3.1. Kv1.3 and KCa3.1 Expression of CAR T Cells

**Figure 1:**
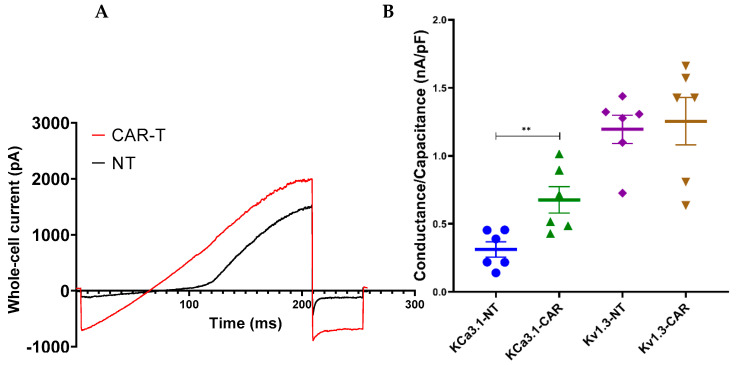
Kv1.3 and KCa3.1 level of CAR T cells. (**A**) Whole-cell current recorded in non-transduced (NT) T cell (black line) and a 3rd-generation CAR T cell (red line) using 200 ms long voltage-ramp protocol ranging from –120 mV up to +50 mV; holding potential was −70 mV. Note the steeper slope of the linear part of the trace for 3rd-generation CAR T cell (higher KCa3.1 conductance expression compared to the NT control). (**B**) Scatter plot of Kv1.3 and KCa3.1 capacitance-normalized conductance of the 3rd-generation CAR transduced and NT cells (thick horizontal line: mean, error bars: SEM, n ≥ 6 (donor number), N ≥ 5 (number of cells/donor), and **: *p* < 0.01).

The introduction of the CAR construct into T cells can affect the ion channel expression in the cell membrane. This can definitely modify the Ca^2+^-dependent cellular processes in the T cells. Hence, first, we tested if the ion channel level of the CAR T cells, KCa3.1 and Kv1.3, were influenced by the transduction of the specific CAR protein. Using the patch clamp technique, we evaluated the expression level of these two channels in the third-generation CAR T cells and the control cells (non-transduced or NT T cells). We applied the ramp protocol (with 1 µM free Ca^2+^ in the pipette solution) to record the KCa3.1 and Kv1.3 channels’ current simultaneously as shown in Figure 1A. The KCa3.1 level is reflected by the slope of the curve, where the Kv1.3 channels are not activated (more negative than −40 mV), while the peak current of + 50 mV, at the end of the ramp, after the subtraction of the KCa3.1 current reports the magnitude of the Kv1.3 current. Through this protocol, we determined the expression level of these two channels in the control/non-transduced (NT) and CAR T cells: Figure 1B shows that the KCa3.1 whole-cell, capacitance-normalized conductance level was higher in the third-generation CAR T cells as compared to the NT T cells (0.31 nS/pF for NT vs. 0.64 nS/pF for CAR T cells; *p* = 0.009). In contrast, the Kv1.3 level did not show any difference compared to the NT cells (1.19 nS/pF for NT vs. 1.25 nS/pF for CAR T cells; *p* = 0.77)

### 3.2. CD8 and CD4 CAR T Cells Have Different KCa3.1 but Not Kv1.3 Level

**Figure 2:**
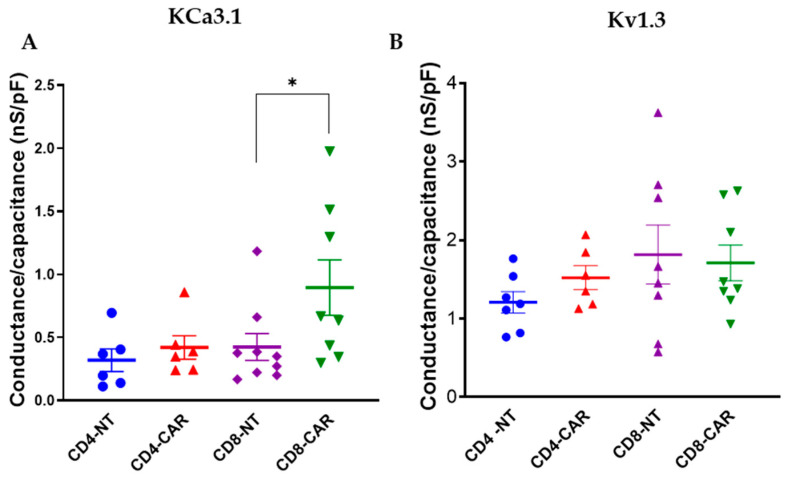
KCa3.1 channel capacitance-normalized conductance is higher in CD8^+^ 3rd-generation CAR T cells. (**A**) KCa3.1 conductance normalized to capacitance in CD4 and CD8 NT and 3rd-generation CAR T cells. (**B**) Kv1.3 capacitance-normalized conductance of NT and 3rd-generation CD4 and CD8 CAR T cells. Thick horizontal line: mean, error bars: SEM, each symbol: mean value for a donor, n ≥ 5 (number of donors), N ≥ 5 (number of cells per donor), and *: *p* < 0.05.

The third-generation CAR T cells had a high expression of KCa3.1 compared to the control, whereas the expression of Kv1.3 was the same. Next, we sought to evaluate if there is a difference in the expression of the ion channels, KCa3.1 and Kv1.3, between the CD4^+^ and the CD8^+^ CAR T cells, since the killing efficiency is different in these two groups. Hence, we evaluated the KCa3.1 and Kv1.3 conductance in these T cell subsets. We applied the same protocol as detailed above, except we plated the cells with the antibody adhesion method to select the CD4^+^ or CD8^+^ subpopulation. We found that the CD8^+^ CAR T cells had a higher KCa3.1 expression level as compared to the CD8 NTs (0.42 nS/pF for CD8^+^ NT and 0.895 nS/pF for CD8^+^ CAR T cells; *p* = 0.04); however, no differences in the KCa3.1-normalized conductance of the CD4^+^ cells and Kv1.3 expression for all the groups were found (Figure 2).

### 3.3. Ca^2+^ Response of CD8^+^ CAR T Cells Is Suppressed

**Figure 3:**
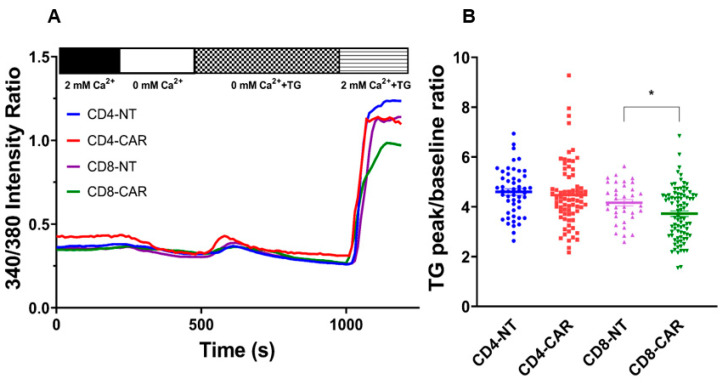
Cytosolic Ca^2+^ response of CD4^+^ and CD8^+^ 3rd-generation CAR T cells. (**A**) The representative traces are the average of 18 CD4^+^ NT T cells (blue), 20 CD4^+^ CAR T cells (red), 12 CD8^+^ NT cells (purple) and 14 CD8^+^ CAR T cells (green). (**B**) Scatter plot of Ca-fold change (ratio measured after addition of 2 mM Ca^2+^ over before addition of 2 mM Ca^2+^ in the presence of TG) in 3rd-generation CAR and NT, CD4^+^ and CD8^+^ cells; each point represents a cell. *: *p* < 0.05.

As we mentioned above, the KCa3.1 and Kv1.3 channels maintain the negative membrane potential that is essential for the driving force of the Ca^2+^ influx through CRAC channels. The activation of the CRAC triggers different pathways responsible for the effector functions of T cells. Hence, the thapsigargin (TG)-evoked Ca^2+^ response of third-generation CD4^+^/CD8^+^ CAR and NT T cells were assessed by the measurements of the cytosolic Ca^2+^ changes using FURA-2 ratiometric imaging. Figure 3A displays the time course of the Ca^2+^ measurement: First, the cells were bathed in 2 mM Ca^2+^ solution and then the extracellular solution was changed for the absolute 0 mM Ca^2+^ (this first step serves to test the integrity of the cell membrane and the viability). Afterwards, 1 µM of TG dissolved in Ca^2+^-free solution was added to the cells to empty the intracellular Ca^2+^ store (ER), which triggers the assembly of STIM1 and ORAI1. Following that, the Ca^2+^ flow through the pore formed by ORAI1 and activated by STIM1 could be detected upon re-addition of the 2 mM Ca^2+^ including the 1 µM TG. The averaged traces in Figure 3A exhibit that all T cell types have the same basic Ca^2+^ level unlike the Ca^2+^ response upon activation of CRAC and re-addition of extracellular Ca^2+^. We found that the Ca^2+^ response of NT CD8^+^, NT CD4^+^, and third-generation CD4^+^ CAR cells were similar, while the CD8^+^ CAR cells had a significantly lower response compared to the control cells. The 2 Ca^2+^/0 Ca^2+^ ratio (peak over baseline) in Figure 3B, which reports on the CRAC expression in the cells, was significantly reduced in the third-generation CD8^+^ cells compared to the CD8^+^ NT control (*p* = 0.02). These observations may suggest that the Ca^2+^ influx through the CRAC channel is affected by the presence of the CAR in the membrane and might have an impact on their killing potential.

### 3.4. Kv1.3 and KCa3.1 Suppression Facilitates Killing Capacity of CD8^+^ CAR T Cells

**Figure 4:**
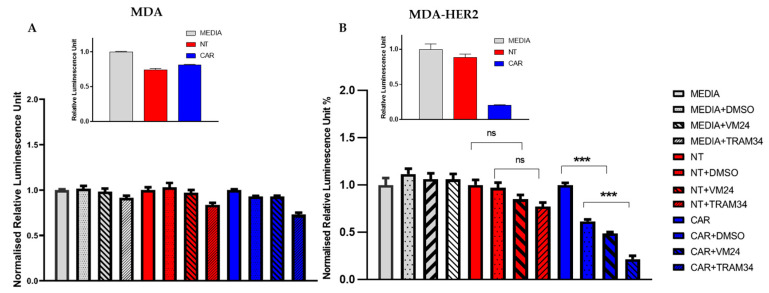
KCa3.1 and Kv1.3 suppression enhances target cell elimination of CAR T cells. Firefly luciferase-based cytotoxicity assay using HER2-speficic CAR T cells, (**A**) MDA (HER2 negative) and (**B**) MDA-HER2 (HER2^+^), as targets at 1:1 ratio (E:T) (n = 4); the assay was performed in triplicates. Vm24 was applied at 1 nM, and TRAM-34 concentration was 1 µM. The intensity values in each group (gray (MEDIA) only target cells (MDA (left), MDA-HER2 (right)) were plated; red (NT): target cells with CD8^+^ NT cells were co-cultured; and blue (CAR): target cells and CD8^+^ CAR T cells were plated) were normalized to the first column of the group (grey to the MEDIA, red to the NT, and blue to the CAR) to emphasize the relative change within the group. Insert figures in both panels show the media-normalized luciferase intensities to visualize the killing capacity of CAR T cells. ***: *p* < 0.001; ns: not significant.

**Figure A1:**
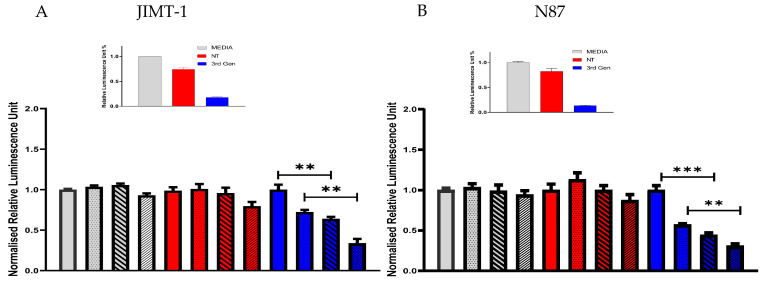
KCa3.1 and Kv1.3 suppression enhances target cell elimination of CAR cells. Firefly luciferase-based cytotoxicity assay using HER2-speficic CAR T cells, (**A**) MDA (HER2 negative) and (**B**) MDA-HER2 (HER2^+^), as targets at 1:1 ratio (E:T) (n = 4); the assay was performed in triplicates. Vm24 was applied at 1 nM, and TRAM-34 concentration was 1 µM. The intensity values in each group (gray (MEDIA) only target cells (MDA (left) and MDA-HER2 (right)) were plated; red (NT): target cells with CD8^+^ NT cells were co-cultured; and blue (CAR): target cells and CD8^+^ CAR T cells were plated) were normalized to the first column of the group (grey to the MEDIA, red to the NT, and blue to the CAR) to emphasize the relative change within the group. Insert figures in both panels show the media-normalized luciferase intensities to visualize the killing capacity of CAR T cells. **: *p* < 0.01, ***: *p* < 0.001; ns: not significant.

CD8^+^ cytotoxic cells may have an important role in the elimination of tumor cells, in which the K^+^ ion channels participate via shaping the Ca^2+^ signaling [25]. Next, we investigated the target cell killing activity of HER2-specific CD8^+^ CAR T cells upon application of Vm24 (specific blocker of Kv1.3, 1 nM) and TRAM-34 (KCa3.1 antagonist, 1 µM) cells in a firefly luciferase (ffLuc) activity-based cytotoxicity assay at a 1:1 of E: T ratio by using ffLuc-modified MDA-HER2, JIMT 1, and N87 target cells. MDA cells not expressing HER2 served as the HER2-negative controls. Our results show that CAR T cell populations recognized and killed HER2^+^ target cells (Figure 4B), while NT T cells had no cytolytic activity confirming specificity (Figure 4A). Upon the addition of the Kv1.3 inhibitor Vm24, the target cell killing capacity of the CD8^+^ CAR T cells enhanced significantly regardless of the target cell type (MDA-HER: ca. 51%, JIMT-1: ca. 36%, and N87: ca. 55% drop in luciferin intensity; Figure 4B (*p* = 0.0004) and Figure A1). Also, the addition of TRAM-34 (the KCa3.1 blocker) improved the short-term killing efficacy of the CAR T cells in vitro against the HER2^+^ monolayer target cell cultures for each cell line tested here (MDA-HER2: ca. 65%, JIMT-1: ca. 53%, and N87: ca 46% decrease in luciferin intensity; Figure 4B (*p* = 0.0002) and Figure A1).

## 4. Discussion

CAR T cell immunotherapy has been a breakthrough in the last few years. Despite the remarkable clinical achievements, the optimally engineered CAR cell construct remains unsolved. Patients receiving the treatment may suffer from life-threatening toxicities while the low efficacy against solid tumors is an obstacle to overcome [26]. Ion channels of T cells (e.g., Kv1.3, KCa3.1, and CRAC) have been shown to play an important role in regulating multiple cellular functions. The inhibition or knock-down/out of these channels hinder the proliferation and effector function of T cells [27]. However, little is known about the role of these ion channels in anti-tumor immunity to eliminate cancer cells, especially in CAR T cells.

In the present study, we investigated the functional expression of Kv1.3 and KCa3.1 in the third-generation CAR T cell specific to the HER2 protein expressed in certain breast cancer cell types. First, we determined whether the expression of the CAR in the membrane of human T cells would provoke a change in the whole-cell conductance of these two channels. Our findings revealed that the conductance of KCa3.1 of the third-generation CAR T cells was higher than that of the control cells, whereas the Kv1.3 conductance was the same. These results clearly showed that this ion channel expression is not suppressed by the introduction of CAR into the CD3^+^ cells, so the effector function, as well as the Ca^2+^-dependent signaling, could not be modified due to the vast change in these channels’ expression. Furthermore, the increased activity of KCa3.1 can contribute to the chemokine-driven migratory capacity of CAR cells, which could facilitate their infiltration into the tumors [28].

**Figure A2:**
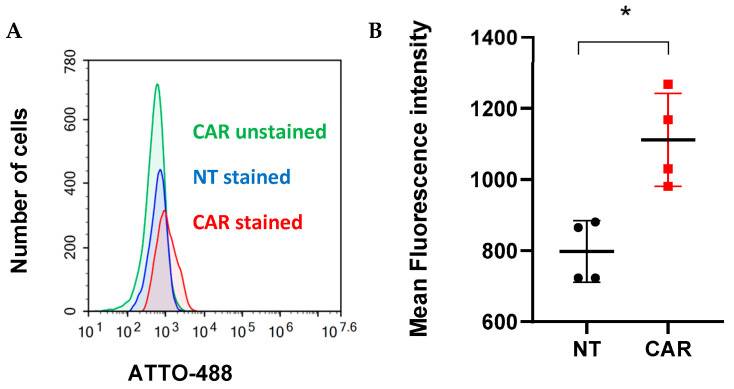
KCa3.1 expression of CD8^+^ CAR T cells. (**A**) Mean fluorescence intensity of KCa3.1 channels in CD8^+^ CAR and NT T cells using mouse anti KCa3.1-ATTO-488 antibody. *: *p* < 0.05. (**B**) CD8^+^ NT and CAR T cells (1 × 10^6^) were collected and stained live using ATTO-488 conjugated mouse anti-human KCa3.1 antibody (Alomone Labs, cat #: ALM-051-AG). To test CD8 positivity, we used anti-human mouse CD8-APC antibody (Biolegend, cat#: 344721). Histogram represents the double-stained cells with unstained control. Cells were gated on CD8^+^ population and then plotted in the histogram: blue (NT) stained with anti-CD8-APC and anti-KCa3.1-ATTO-488, green (CAR) unstained, and red (CAR) stained with anti. CD8-APC and KCa3.1-ATTO-488. For the labeling, we followed the manufacturers’ protocol.

It is widely accepted that the CD8 ^+^ CAR T cells have a higher killing potential due to their higher lytic activity [29]. Hence, we assessed the ion channel expression in CD4^+^ and CD8^+^ third-generation CAR T cells: the CD8^+^ population of CAR T cells had significantly higher KCa3.1 levels, while CD4^+^’s had no significant change in the expression of both KCa3.1 and Kv1.3 as compared to the corresponding NT control group (Figure 2A and Figure A2). However, we should mention that the KCa3.1 conductance was also elevated for CD4^+^ CARs as compared to NT cells, though statistically it was not significant. Previously, it was also reported that KCa3.1 in CD8^+^ T cells plays a role in chemokine gradient-evoked migration [28], which could guide them to the tumor site. We think that this increased activity of KCa3.1 could facilitate the entry of CD8^+^ CAR T cells into solid tumors and lead to a successful therapeutic outcome. It has also been shown that the anti-tumor cytotoxicity of CAR T cells is related to the cytokine/chemokine profile [19]. We think that ion channels might have a role in these activated pathways. This is why it is important to perform further investigations.

Next, we investigated the Ca^2+^-response in CAR T cells: the cytosolic Ca^2+^ level is considered to be one of crucial triggers in controlling a wide range of effector functions. Ca^2+^ influx through the CRAC channel is regulated by the concerted activity of ion channels in the T cell membrane; hence, we chose thapsigargin-evoked activation of CRAC, which bypasses the TCR/CD3 activation pathway and provides us with direct proof of ion channel regulation of the Ca^2+^ response. In our current study, we unveiled whether the expression of the anti-HER2 CAR will influence the Ca^2+^ response. Our result shows that in the case of CD8^+^ CAR T cells, the Ca^2+^ influx is reduced as compared to the CD8 NT cells. On the contrary, CD4^+^ CAR and NT T cells displayed the same Ca^2+^ signaling provoked by the Ca^2+^ release from the ER. Since the expression of KCa3.1 was higher in CD8 CARs, which could lead to enhanced SOCE, we suppose that the expression of CRAC (ORAI1 and STIM1) should be lower in CD8^+^ CAR T cells.

Finally, we tested if the blockage of ion channels (Kv1.3 and KCa3.1) can influence the target cell killing efficacy of CD8^+^ CAR T cells. Previous studies demonstrated that TRAM-34 inhibits the native and cloned KCa3.1 channel in human T lymphocytes with a K_d_ of 20–25 nM and that is 200- to 1500-fold selective over other ion channels [30], while Vm24 with a K_d_ value of ca. 3 pM is highly selective for Kv1.3 channels [31]. Here, we obtained that reduced conductance of Kv1.3 channels (when almost every channel is blocked (app. 300-fold K_d_ of Vm24 was applied)) improved the killing efficiency of the CAR T cells. Moreover, the antagonism of the KCa3.1 channels also facilitated the HER2^+^ cell elimination. Though, logically, the blocking of KCa3.1 and Kv1.3 channels should imply some impairment of CTL function, this straightforward scenario may not work for, e.g., CTLs and NKs. Previously, it was shown that blocking KCa3.1 using TRAM-34 in natural killer (NK) cells induces depolarization of the plasma membrane, which improves the cytotoxicity and degranulation toward their target cells. However, it did not affect their ability to migrate and their expression of chemokines [32]. Recent studies have shown that the reduction in the calcium entry (SOCE) by specific inhibition of KCa3.1/Kv1.3 channels enhances the cytotoxic activity of NK cells against the T-ALL Jurkat cell line [33]. It was also reported that efficient target cell killing requires an optimal intracellular Ca^2+^ concentration, which could be effectively attained by impairing the functional expression of CRAC, even by means of the partial downregulation of ORAI1 [34]. Since there exist highly potent and selective blockers, or even molecular biology tools to regulate the expression for these K^+^ channels, we suppose their modification could open up a new modality in CAR T cell therapy.

We must mention that our study has limitations. First, the signaling pathway, which could be rewired upon CAR introduction, still needs to be investigated to understand the effect of KCa3.1 expression on, e.g., cytokine productions and migration. Second, the efficacy of these channel blockers should be evaluated in vivo, mainly due to the suppressive behavior of the tumor microenvironment. However, we think that our results provide functional information on major ion channels of T cells in their engineered “foster siblings” and can be applied for future research focusing on improving CAR T cell therapy.

## 5. Conclusions

In summary, we could show that (1) the ion channels of T cells necessary for Ca^2+^-related functions are also present in HER-CAR-expressing T cells, (2) CD8^+^ CAR T cells have a higher KCa3.1 but lower CRAC level as compared to the control, and (3) the inhibition of Kv1.3/KCa3.1 channels facilitates the cancer cell elimination capacity. Based on these results, we propose that the modulation of ion channels in CAR T cell therapy could be novel approach to achieve an improved outcome.

## Data Availability

We did not analyze public datasets in this manuscript.
